# Air Pollution and Respiratory Hospital Admissions in Kuwait: The Epidemiological Applicability of Predicted PM_2.5_ in Arid Regions

**DOI:** 10.3390/ijerph19105998

**Published:** 2022-05-15

**Authors:** Soad Albahar, Jing Li, Mustafa Al-Zoughool, Ali Al-Hemoud, Janvier Gasana, Hassan Aldashti, Barrak Alahmad

**Affiliations:** 1 Environmental and Occupational Health Department, College of Public Health, Kuwait University, Shadadiya 13110, Kuwait; mustafa.alzoughool@ku.edu.kw (M.A.-Z.); janvier.gasana@ku.edu.kw (J.G.); b.alahmad@g.harvard.edu (B.A.); 2 Institute of Child and Adolescent Health, School of Public Health, Peking University, Beijing 100871, China; 3 Environment and Life Sciences Research Center, Kuwait Institute of Scientific Research, Kuwait City 13109, Kuwait; ahomood@kisr.edu.kw; 4 Meteorological Department, Directorate General of Civil Aviation, Kuwait City 13001, Kuwait; calmwind22@hotmail.com; 5 Environmental Health Department, Harvard T.H. Chan School of Public Health, Harvard University, Boston, MA 02115, USA

**Keywords:** desert dust, time series, respiratory admission, Kuwait

## Abstract

Dust is a major component of fine particulate matter (PM_2.5_) in arid regions; therefore, concentrations of this pollutant in countries such as Kuwait exceed air quality standards. There is limited understanding on the impact and burden of high PM_2.5_ concentrations on morbidity in these countries. In this study, we explore the association of PM_2.5_ and the risk of respiratory hospital admissions in Kuwait. A time-series regression model was used to investigate daily variations in respiratory admissions and PM_2.5_ concentrations from 2010 to 2018. Due to the lack of historical air quality sampling in Kuwait, we used estimated daily PM_2.5_ levels from a hybrid PM_2.5_ prediction model. Individual and cumulative lag effects of PM_2.5_ over a 5-day period were estimated using distributed lag linear models. Associations were stratified by sex, age, and nationality. There were 218,749 total respiratory admissions in Kuwait during the study period. Results indicate that for every 10 μg/m^3^ increase in PM_2.5_, a 1.61% (95% CI = 0.87, 2.35%) increase in respiratory admissions followed over a 5-day cumulative lag. Our estimates show that a 10 μg/m^3^ reduction in average exposure will potentially avert 391 yearly respiratory admissions (95% CI = 211,571), with 265 fewer admissions among Kuwaitis (95% CI = 139,393) and 262 fewer admissions among children under 15 years of age (95% CI = 125,351). Different strata of the Kuwaiti population are vulnerable to respiratory hospitalization with short-term exposure to PM_2.5_, especially those under 15 years of age. The findings are informative for public health authorities in Kuwait and other dust-prone countries.

## 1. Introduction 

Kuwait is well known for its desert climate, with dry, hot summers, scarce vegetation, and strong winds [1]. Countries with such unique weather conditions are prone to a considerable amount of dust and dust storms [2,3]. Because dust is a major component of ambient particulate matter with aerodynamic diameter less than 2.5 microns (PM_2.5_) in the region, concentrations of PM_2.5_ in Kuwait far exceed the World Health Organization (WHO) 24 h (15 μg/m^3^) and annual (5 μg/m^3^) air quality standards [4,5].

Exposure to particulate matter is associated with detrimental health effects such as mortality [6,7] and low birth weight in newborns [8,9]. 

Several studies found significant increases in the risk of respiratory hospital admissions across all age groups associated with high PM_2.5_ [10,11,12,13,14]. Those with pre-existing respiratory conditions such as asthma are more susceptible, with an increase in asthma admissions associated with increases in PM_2.5_ concentrations [11,15,16]. The extent to which we understand the impact and burden of high PM_2.5_ concentrations on morbidity in the country is limited. 

To date, only two studies have assessed the health impacts of particulate pollution in Kuwait. The first, investigated the effects of particulate matter from dust storms on respiratory-related admissions in hospitals in Kuwait from January 1996 to December 2000 [17]. The study, however, only looked at exposure to particulate matter with aerodynamic diameter less than 10 microns (PM_10_). PM_2.5_ is rather more harmful than PM_10_ because fine particles can penetrate the lungs’ small airways and reach the alveoli as compared to large and coarse particles, which cannot penetrate deep into the lungs [18]. The second study assessed exposure to PM_2.5_ and its impact on cardiovascular and respiratory disease mortality and morbidity. The findings suggest that the proportion of excess cases of respiratory diseases attributable to PM_2.5_ exposure was higher than that of cardiovascular diseases [19]. PM_2.5_ exposure is shown to be a risk factor for respiratory disease morbidity in the country. 

New studies are warranted to investigate the harmful effects of PM_2.5_ to contribute to the country’s environmental policies on air quality health assessments due to the ubiquitous nature of dust in the region and the associated regulatory challenges. Since there is a lack of historical air quality sampling and ground monitoring stations in Kuwait, assessment of the health impact of particulate matter exposure is lacking. To address this, we used PM_2.5_ prediction models to estimate average daily PM_2.5_ levels. According to Jing et al. [20], this novel approach can be used in regions with limited monitoring networks. In this study, we examined the association between predicted PM_2.5_ and the risk of respiratory-related hospital admissions among different strata of the Kuwait population.

## 2. Materials and Methods

### 2.1. Admissions Data 

Upon ethical approval from the Ministry of Health, we obtained daily respiratory-related hospital admissions data in Kuwait across 17 public hospitals for the period from 1 January 2010 to 31 December 2018, from the National Center for Health Information, Department of Vital Statistics, Ministry of Health, Kuwait. The cause of admission was classified based on the international classification of diseases version 10 (ICD-10) codes; J00–J99 used for total respiratory admissions and J45 for asthma admissions.

### 2.2. Environmental Data 

We estimated daily PM_2.5_ levels in micrograms per cubic meter (μg/m^3^) using a hybrid PM_2.5_ prediction model. The detailed methodology can be found in Li et al. [20]. In brief, using machine learning methods and generalized additive mixed models combining visibility, satellite retrievals of aerosol optic depth, land use data, and ground-based observations, the model predicted PM_2.5__levels_ in Iraq and Kuwait at a high spatial (1 × 1 km) and temporal (daily) resolution [20]. The model was evaluated with the 10-fold cross validation with an R^2^ value of 0.71. For each day, PM_2.5_ was averaged from all urban pixels in the country. The exposure data are available from the corresponding author (JL) upon reasonable request. Meteorological variables such as daily average temperature (°C) and relative humidity (%) were obtained from the Meteorological Department of the Directorate General of Civil Aviation for the same study period. 

### 2.3. Study Design & Statistical Analysis

To assess the association between daily counts of respiratory admissions and daily PM_2.5_ exposure, we applied generalized linear models (GLMs) for a time-series structured data using Quasi-Poisson regression to account for overdispersion. The linearity of PM_2.5_ was initially assessed in generalized additive models (GAMs) by applying penalized splines. We then fitted a linear term for PM_2.5_ in the GLMs. Time was modeled with natural splines (7 degrees of freedom per calendar year) and a categorical variable for the day of the week [21,22]. To account for potential delayed effects, 3- and 5-day moving average were used for PM_2.5_. We also fitted distributed lag linear models (*DLM*) to estimate individual and cumulative lag effects of PM_2.5_ over a 5-day period. Temperature was modeled using distributed lag nonlinear models (DLNM); a natural spline with 3 degrees of freedom for the temperature exposure and a 7-day lag period and natural spline with 3 degrees of freedom equally spaced in the log scale for the lag basis. Relative humidity was modeled with natural spline with 3 degrees of freedom. The final model can be described as follows:

(1) $$\log\left(E\left[Y_{i}\right]\right)\;=\;\mathrm{intercept}\;+\;{PM}_{2.5i,l}\;+\;T_{i,t}\;+\;ns\left(RH_{i},\;df\;=\;3\right)\;+\;DOW_{i}\;+\;ns\left(time_{i},\;df\;=\;7\;per\;year\right)$$

where *E*[*Y*_*i*_] is the expected count of total respiratory admissions on day *i*, *PM*_2.5_ refers to daily mean ambient *PM*_2.5_ concentrations with *l* moving average lag days, *T* is the temperature DLNM cross-basis function for *t* lag days, *RH* is relative humidity, *DOW* is day of the week, *ns* is a natural spline, and *df* is degrees of freedom.

We also estimated the burden of hospital admissions from PM_2.5_ exposure by calculating the potential admissions averted for every 10 μg/m^3^ reduction. We used the approach presented by Dominici, et al. [23] and Krishna, et al. [24], where attributable averted admissions are defined as (exp (β × ∆x) − 1) × N, where β being the coefficient for a 1 μg/m^3^ increase in PM_2.5_, ∆x being 10 μg/m^3^, and N being the total number of admissions in a defined time period. In an additional analysis, we investigated the impact of PM_2.5_ on asthma-only admissions. Analyses were carried out using R statistical software version 4.0.3 (R Foundation for Statistical Computing, Vienna, Austria) and *dlnm* package version 2.4.6.

## 3. Results

### 3.1. Summary Statistics

Over the 3294 days between 1 January 2010 and 31 December 2018, there were 218,749 total respiratory admissions in Kuwait. The highest mean admissions per day were found among males, Kuwaitis, and those under the age of 15. The median respiratory admissions per day were 66 (interquartile range [IQR] = 35) and the median PM_2.5_ exposure was 44 μg/m^3^ (IQR = 15.09). Summary statistics of all variables are provided in Table 1. 

### 3.2. Regression Results

Table 2 shows the percentage increase in admission for every 10 μg/m^3^ increase in PM_2.5_ exposure for same-day exposure up to 5 days after exposure. 

**Figure 1:**
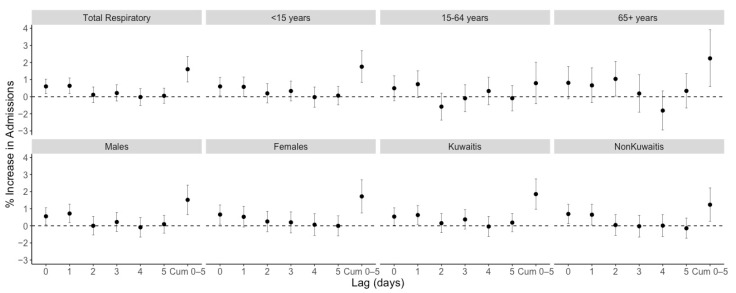
Associations between delayed respiratory admission and PM_2.5_ exposure among different subgroups for distributed lag models up to 5 days lag. All models were adjusted for ambient temperature, relative humidity, long-term trends, and day of the week. Cum; cumulative.

Table 3 and Figure 1 present increased admissions among the various strata examined over 5-day cumulative distributed lags. Overall, we observed a 0.6% (95% confidence intervals [CI] = 0.17, 1.03%) increase in respiratory admissions with every 10 μg/m^3^ increase in same-day PM_2.5_ exposure and a 1.61% (95% CI = 0.87, 2.35%) increase over a 5-day cumulative distributed lag. Moving average lagged effects for 3- and 5-days were significant, showing an increase in total admission of 1.32% (95% CI = 0.77, 1.87%) and 1.38% (95% CI = 0.7, 2.05%), respectively. Although the confidence intervals overlap, there was some heterogeneity in effect size across age groups with every 10 μg/m^3^ increase in exposure contributing to a 1.76% (95% CI = 0.84, 2.68%) increase in admissions among those under 15 years of age, and a 2.24% (95% CI = 0.6, 3.92%) increase in those above 65 years of age. The risk estimates were not statistically significant for the 15–64 age group. A significant association was observed in both males and females, with each 10 μg/m^3^ exposure increment contributing to 1.52% (95% CI = 0.66, 2.39%) and 1.72% (95% CI = 0.76, 2.69%) increase in admissions, respectively. Furthermore, Kuwaitis showed a higher risk of admission than non-Kuwaitis in total. 

Based on our in-depth subgroup analyses, stratifying the subpopulations by nationality, then by sex, then by age (Table 4), we found that Kuwaiti female children had the highest effect estimates. Non-Kuwaiti males showed a higher RR of admission than Kuwaiti males in total. Although nonsignificant, non-Kuwaiti females had the lowest risk of admission. As shown in Figure S1, Tables S1 and S2, results for asthma admissions were associated with a wide uncertainty (Supplementary Materials).

### 3.3. Admissions Averted by Reducing Exposures

Table 5 shows the total respiratory admissions that can be averted for a 10 μg/m^3^ reduction in PM_2.5_ exposure in Kuwait. Our estimates show that a 10 μg/m^3^ reduction in average exposure may potentially reduce respiratory admissions by 391 (95% CI= 211, 571) patients each year. 

## 4. Discussion

In this study, we were able to leverage statistical models to obtain a spatiotemporally resolved PM_2.5_ over a historical period in Kuwait that otherwise did not exist. We used this dataset to investigate the associations with respiratory-related hospital admissions. We estimate that a total of 391 respiratory admissions each year can be averted if PM_2.5_ levels in the country drop by 10 μg/m^3^. Overall, for every 10 μg/m^3^ increase in PM_2.5_, there is a 1.61% increase in total respiratory admissions over a 5-day cumulative distributed lag. Different strata of the Kuwait population are vulnerable to PM_2.5_ exposure, especially the elderly and those under 15 years of age. 

Differences in PM_2.5_ impacts on the Kuwaiti population by age, sex, and nationality were investigated to determine the vulnerability of the population. Those aged 65 and over were especially susceptible to PM_2.5_ exposure, followed by those under 15 years of age. The strength of these associations is consistent with studies showing stronger impacts of PM_2.5_ on respiratory admissions among the elderly [25]. Adverse impacts on the elderly are expected based on the literature and are likely related to weakened immune system and decreased lung function within this age group [26,27,28]. It is also important to note that respiratory diseases such as pneumonia, chronic obstructive pulmonary disease (COPD), and chest infections are more prevalent in this older age group, and PM_2.5_ exposure is likely contributing to larger effect estimates in the presence of these diseases [29,30,31]. The other significant association was seen among the <15 age group, which is expected given that children’s lungs are not fully developed. Children breathe more air relative to body size than adults and are likely to spend more time outdoors, increasing their exposure to pollutants [32]. With regard to sex, a study exploring the differential impact of PM_2.5_ on respiratory outcomes for males and females in different counties in the USA found that PM_2.5_ exposure was associated with a higher relative increase in hospitalization for respiratory tract infections and asthma in women than in men [11,33]. Similarly, Shakerkhatibi, et al. [34] reported that the odds ratio for females was 1.5 times higher than males for risk of respiratory admission due to PM_2.5_ exposure. We found Kuwaiti females to have a high risk of admission compared to males. The exact reason for these differences is unclear, but could be attributed to physiological, behavioral, and/or psychosocial determinants of health [35]. Possible differences in lifestyle between the sexes could result in differences in exposure patterns and, thus, result in this disparity [35]. The pathways through which PM_2.5_ exposure affects the health of men and women differently needs to be further explored.

As expected, non-Kuwaiti males exhibited a large relative risk of admissions. Non-Kuwaiti adult males are more vulnerable to higher ambient pollutant exposure given the outdoor nature of many migrant worker jobs such as construction and sanitation [36]. This is further supported by a previous study conducted in Kuwait on the impact of poor air quality on mortality that reported an increase in non-Kuwaiti male mortality rates associated with exposure to air pollution and dust storms [37]. This study, however, used visibility, a binary outcome, to describe adverse air quality, whereas in our study we used continuous PM_2.5_, which is more quantifiable and can provide better regulatory inference and allow for comparison across different studies. Our finding could be influenced by social factors related to access to healthcare, where Kuwaitis can utilize public hospital services for free, whereas non-Kuwaitis must pay a fee to be admitted and/or receive treatment. This likely played a role in potentially underestimating the risk of respiratory hospital admissions among the non-Kuwaiti subgroup. In Thalib and Al-Taiar’s [17] paper investigating the impact of dust storms on respiratory hospital admissions in Kuwait, binary measures for exposure were employed (dust storm vs. non-dust storm days) for the period between 1996 to 2000. Our study used continuous PM_2.5_ measures instead and provided an analysis on hospital admissions using recent data from the past decade.

Overall, our findings are in line with previous studies conducted globally. A study in China reported a respiratory admission relative risk of 1.06 per 10 μg/m^3^ rise in exposure PM_2.5_ at lag 0–2 days [38]. A meta-analysis of 110 peer-reviewed time-series studies assessing associations between PM_2.5_ and daily mortality and hospital admissions concluded that a 10 μg/m^3^ increment in PM_2.5_ is associated with an increase in risk of admission for respiratory diseases by 0.96% [10]. 

Particulate matter exposure impacts health negatively especially among individuals with pre-existing lung conditions. PM inhalation among respiratory patients is associated with pulmonary function decrements where reductions in forced expiratory volume in 1 s (FEV1) and forced vital capacity (FVC) are observed [39,40,41,42]. Research has also demonstrated that increased levels of particulate matter seem to increase symptoms of respiratory distress (i.e., wheezing and shortness of breath) and asthma medication use [32,43,44,45]. Furthermore, pollutants in the air are known to interact with airborne allergens, increasing the risk of atopic sensitization and exacerbation of respiratory disease symptoms [46,47]. When fine PM along with these allergens penetrate deep into the lungs, irritation is caused which induces alveolar inflammation that limits air flow and in turn results in acute respiratory episodes [48]. The impacts of acute PM inhalation pose a threat to the health of the population, especially vulnerable groups. Therefore, assessing the relationship between PM_2.5_ and respiratory outcomes is of optimum importance. 

### Limitations

Based on the literature review, we believe that this is the first study in the region to examine the association of PM_2.5_ with respiratory admissions. However, the study has a few limitations. The use of estimated daily mean PM_2.5_ measurements rather than direct measurements from monitoring stations in Kuwait is potentially associated with exposure measurement error. However, these PM_2.5_ prediction models ensure that there are no days with missing data. Although we believe that the PM_2.5_ prediction models had good predictive ability (R^2^ = 0.71), it is still less preferred than direct ground monitoring readings, as less measurement error in the exposure would occur had we measured PM_2.5_ continuously from ground stations for the study period. Despite this, the predictions are the best available data, since historical ground monitoring in Kuwait does not exist. Additionally, in this study, we assumed that all individuals in the population were exposed to PM_2.5_ at the same level, since we did not have address data for admitted patients. Moreover, for future directions, it would be valuable to examine hospital readmissions to assess their association with exposure to PM_2.5_ in the long-term. Finally, the findings of this study should be interpreted within the local context and population characteristics in Kuwait. 

## 5. Conclusions

The present study is the first to explore the impact of PM_2.5_ exposures on daily respiratory admissions in Kuwait. Our analysis suggests that different strata of the Kuwaiti population are vulnerable to respiratory disease hospitalization when exposed to PM_2.5_ in the short-term, especially the elderly and those under 15 years of age. The results of the study highlight the importance of preventative care for Kuwait’s susceptible population to decrease the risk of respiratory complications during bad weather events. Potential warning systems can be put in place with air quality alerts to protect those who are vulnerable, especially children. In addition, with such information, hospitals would be able to predict increases in admissions and, thus, be better equipped to handle high volumes of emergency visits during days with higher PM levels. These findings contribute to the limited evidence related to the association between health and exposure to dust-dominant ambient PM_2.5_ in Kuwait and in the Middle East. 

## Figures and Tables

**Table 1:** Descriptive statistics of the population and environmental exposures over the study period (1 January 2010 to 31 December 2018).

	Total	Mean	SD	Median	Min	IQR	Max
Cause of Admissions (adm./day)							
Total Respiratory	218,403	66.77	24.84	66	3	35	151
Asthma	22,701	6.99	4.15	6	1	5	50
Sex (adm./day)							
Male	124,622	38.10	14.37	38	1	20	85
Female	93,781	28.67	11.78	28	2	16	76
Nationality (adm./day)							
Kuwaiti	128,533	39.32	15.89	38	1	23	91
Non-Kuwaiti	89,870	27.45	10.73	27	2	14	78
Age groups (adm./day)							
<15	134,191	41.05	18.25	40	2	25	100
15–64	59,655	18.22	7.30	18	1	10	63
65+	24,557	7.51	3.49	7	0	5	25
Exposure							
PM_2.5_ (μg/m^3^)	-	46.93	16.89	44	11.91	15.09	403.80
Average Temperature (°C)	-	27.26	9.84	28	5.70	18.50	43.90
Average relative humidity (%)	-	33.80	20.01	28.50	6.40	32.00	93.20

Total number of days measured = 3294 days. Adm; admissions, SD; standard deviation, Min; minimum, IQR; interquartile range, Max; maximum.

**Table 2:** Increase in total respiratory admission for 10 μg/m^3^ increase in PM_2.5_ exposure for 0–5 lag days using distributed lag models and moving average of 3 and 5 days.

Lag	% Increase in Admissions	95% CI	
		Lo %	Hi %
Distributed Lags			
0	0.60 *	0.17	1.03
1	0.64 *	0.18	1.1
2	0.11	−0.35	0.57
3	0.21	−0.26	0.69
4	−0.02	−0.51	0.47
5	0.05	−0.39	0.5
Cumulative 0–5 (main model)	1.61 *	0.87	2.35
Moving Average Lags			
3	1.32 *	0.77	1.87
5	1.38 *	0.7	2.05

* = statistically significant (Sig. = 0.05). All models were adjusted for ambient temperature, relative humidity, long-term trends, and day of the week. CI; confidence interval.

**Table 3:** Sub-group analysis for the association between average PM_2.5_ and respiratory admissions over 0–5 cumulative days lag period using distributed lag models.

Subgroup	% Increase in Admissions	95% CI	
		Lo %	Hi %
Nationality			
Kuwaiti	1.85 *	0.97	2.75
Non-Kuwaiti	1.24 *	0.27	2.21
Sex			
Male	1.52 *	0.66	2.39
Female	1.72 *	0.76	2.69
Age			
<15 years	1.76 *	0.84	2.68
15–64 years	0.79	−0.42	2.01
65+ years	2.24 *	0.60	3.92

* = statistically significant (Sig. = 0.05). All models were adjusted for ambient temperature, relative humidity, long-term trends, and day of the week. CI; confidence interval.

**Table 4:** In-depth subgroup analysis for the association between average PM_2.5_ and respiratory admissions over 0–5 cumulative days lag period using distributed lag models.

Nationality	Sex	Age	% Increase in Admissions	95% CI	
				Lo%	Hi%
Kuwaiti	Male				
		Total	1.60 *	0.52	2.70
		<15	1.59 *	0.29	2.90
		15–64	1.38	−0.72	3.53
		65+	1.62	−1.28	4.61
	Female				
		Total	2.15 *	0.99	3.32
		<15	2.38 *	0.90	3.89
		15–64	1.27	−0.93	3.53
		65+	2.03	−0.48	4.60
Non-Kuwaiti	Male				
		Total	2.15 *	0.99	3.32
		<15	1.79 *	0.29	3.32
		15–64	0.12	−1.83	2.10
		65+	3.20	−1.01	7.59
	Female				
		Total	0.99	−0.42	2.42
		<15	1.18	−0.58	2.97
		15–64	−0.20	−2.82	2.49
		65+	1.75	−2.46	6.14

* = statistically significant (Sig. = 0.05). All models were adjusted for ambient temperature, relative humidity, long-term trends, and day of the week. CI; confidence interval.

**Table 5:** Respiratory admissions potentially averted for 10 μg/m^3^ reduction in PM_2.5_ exposure in Kuwait.

Group	Total Admissions	Reduction in Number of Admissions per Year for 10 μg/m^3^ Reduction in PM_2.5_ (95% CI)
Overall	218,403	391 (211, 571)
<15	134,191	262 (125, 351)
15–64	59,655	53 (−28, 134)
65+	24,557	62 (17, 107)
Male	124,622	211 (92, 331)
Female	93,781	180 (80, 281)
Kuwaiti	128,533	265 (139, 393)
Non-Kuwaiti	89,870	124 (0, 222)

All models were adjusted for ambient temperature, relative humidity, long-term trends, and day of the week.

## Data Availability

Restrictions apply to the availability of these data. Data was obtained from the Kuwait Ministry of Health and are available, from the corresponding author S.A., with the permission of the Kuwait Ministry of Health.

## Supplementary Materials

The following supporting information can be downloaded at: https://www.mdpi.com/article/10.3390/ijerph19105998/s1, Figure S1: Associations between delayed asthma admission and PM_2.5_ exposure among the total population for distributed lag models up to a 5-day lag. All models were adjusted for ambient temperature, relative humidity, long-term trends, and day of the week. Cum; cumulative. Table S1: Increase in total asthma admission for 10 μg/m^3^ increase in PM_2.5_ exposure for 0–5 lag days using distributed lag models and a moving average of 3 and 5 days. Table S2: Subgroup analysis for the association between average PM_2.5_ and asthma admissions over 0–5 cumulative days lag period using distributed lag models.
