# Supplementary material for: Air Pollution and Respiratory Hospital Admissions in Kuwait: The Epidemiological Applicability of Predicted PM2.5 in Arid Regions
Source: Int J Environ Res Public Health. 2022 May 15;19(10):5998. doi: 10.3390/ijerph19105998 (PMC9140349; doi:10.3390/ijerph19105998)
Supplement: Supplementary file 1 [file ijerph-19-05998-s001.zip › Table S1.pdf]

Lag	% Increase in asthma admissions	95 % CI	
		Lo %	Hi %
Distributed Lags			
0	0.07	-0.98	1.14
1	0.89	-0.20	2.00
2	-0.39	-1.54	0.77
3	0.52	-0.59	1.64
4	0.73	-0.41	1.88
5	-0.84	-1.97	0.30
Cum 0-5	0.97	-0.85	2.82
Moving Average Lags			
3	1.00	-0.35	3.28
5	1.63	0.00	2.05

** = statistically significant (Sig. = 0.05)*
