# Supplementary material for: Air Pollution and Respiratory Hospital Admissions in Kuwait: The Epidemiological Applicability of Predicted PM2.5 in Arid Regions
Source: Int J Environ Res Public Health. 2022 May 15;19(10):5998. doi: 10.3390/ijerph19105998 (PMC9140349; doi:10.3390/ijerph19105998)
Supplement: Supplementary file 1 [file ijerph-19-05998-s001.zip › Table S2.pdf]

	% Increase in asthma admissions	95 % CI	
		Lo %	Hi %
Kuwaiti	1.36	-0.82	3.58
Non-Kuwaiti	0.12	-2.81	3.14
Male	0.73	-1.68	3.20
Female	1.10	-1.36	3.62
< 15 yrs	1.43	-0.83	3.74
15-64 yrs	-0.49	-3.90	3.04
65+ yrs	2.52	-2.22	7.50

** = statistically significant (Sig. = 0.05)*
