# Supplementary figures and images for: Air Pollution and Respiratory Hospital Admissions in Kuwait: The Epidemiological Applicability of Predicted PM2.5 in Arid Regions
Source: Int J Environ Res Public Health. 2022 May 15;19(10):5998. doi: 10.3390/ijerph19105998 (PMC9140349; doi:10.3390/ijerph19105998)

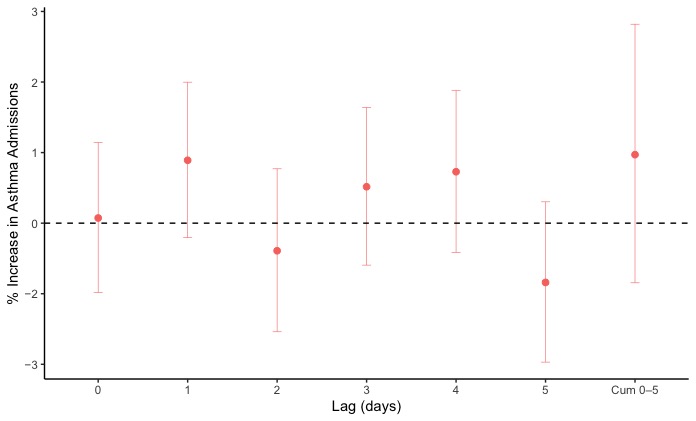

Supplement: Supplementary file 1 [file ijerph-19-05998-s001.zip › Figure S1.jpeg]
